# Supplementary material for: A Novel Iodine–Dextrin Complex Exhibits No Acute or Subacute Toxicity and Enhances Azithromycin Efficacy in an LPS-Induced Sepsis Model
Source: Pharmaceutics. 2025 Aug 11;17(8):1040. doi: 10.3390/pharmaceutics17081040 (PMC12388929; doi:10.3390/pharmaceutics17081040)
Supplement: Supplementary file 1 [file pharmaceutics-17-01040-s001.zip › pharmaceutics-3736931-supplementary.pdf]

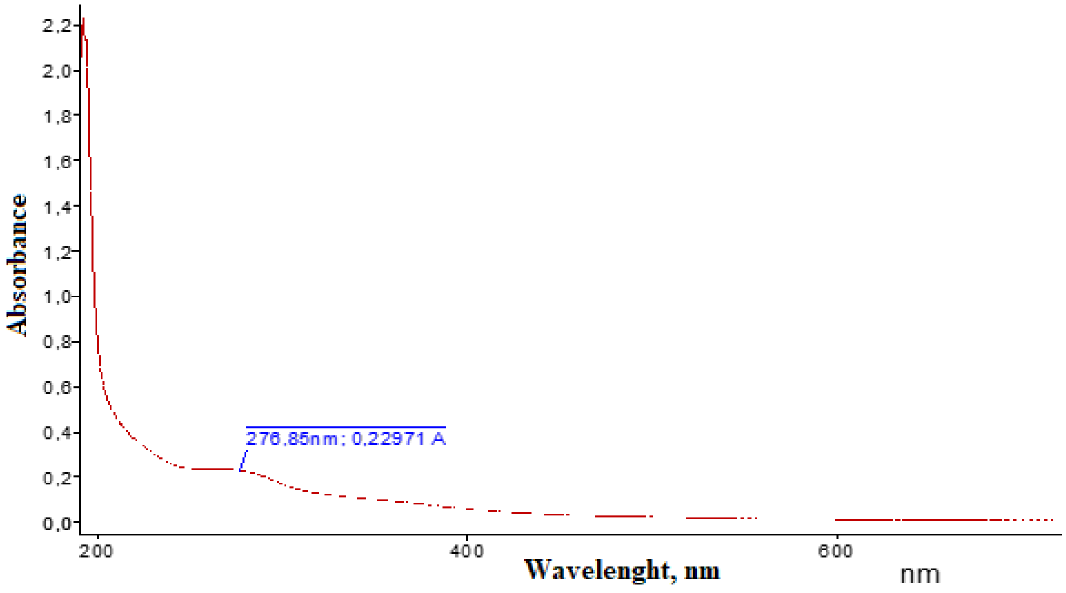

Figure S1 – UV spectrum of dextrin

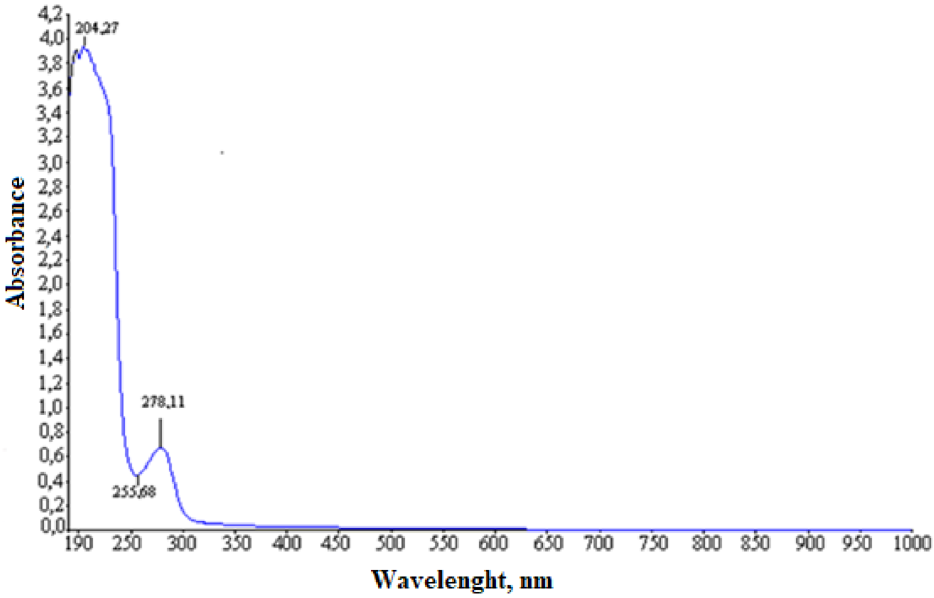

Figure S2 – UV spectrum of albumin

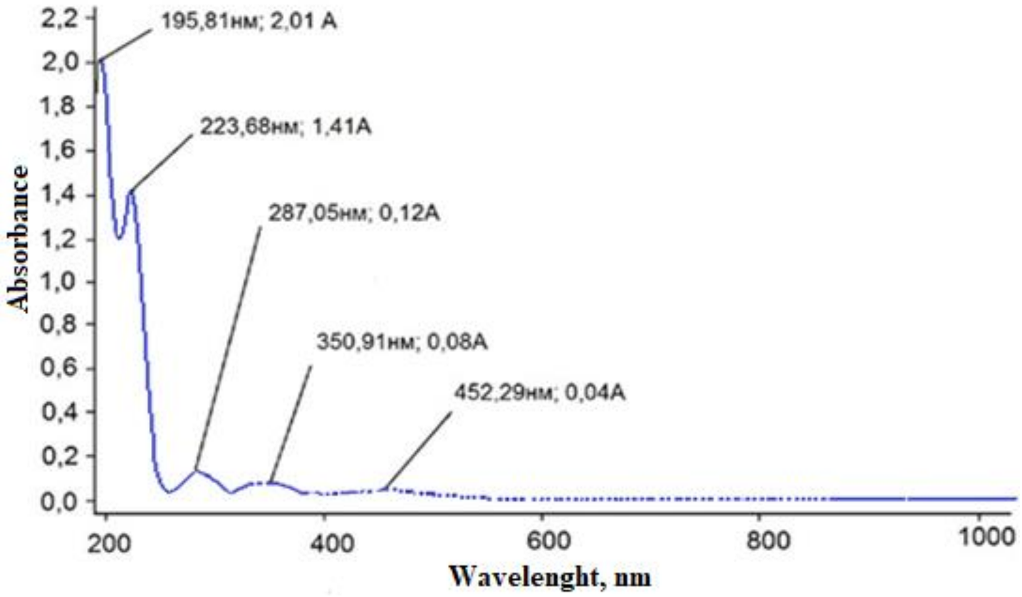

Figure S3 – UV spectrum of an aqueous solution of I_2+KI

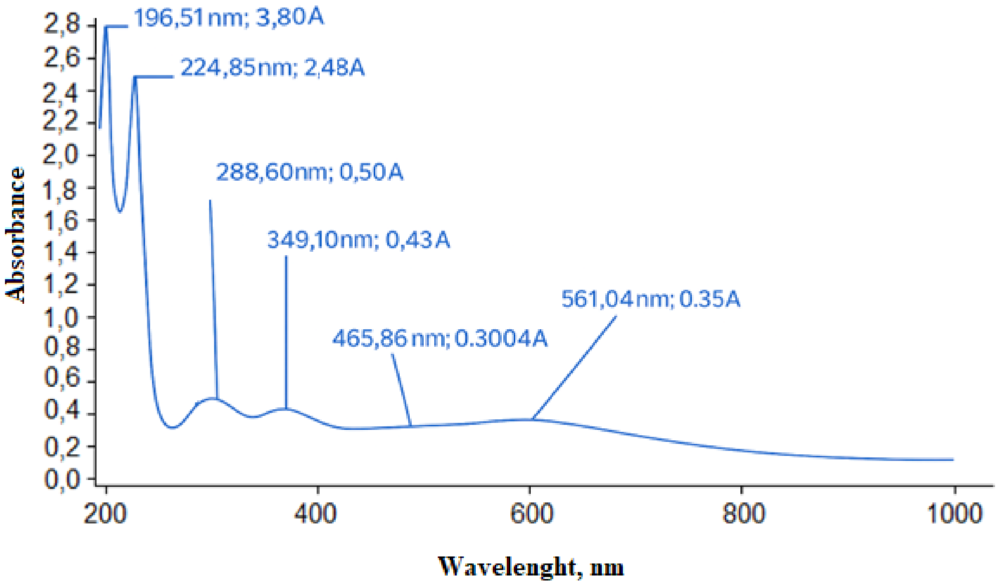

Figure S4 – UV spectrum of iodine-dextrin-based semi-organic complex (PA)
